# Deep Learning Predicts Mutations and Outcomes in Gastrointestinal Stromal Tumors

**DOI:** 10.1158/0008-5472.CAN-26-0132

**Published:** 2026-06-08

**Authors:** Arianna Bonetti, Van-Linh Le, Zunamys I. Carrero, Fabian Wolf, Marco Gustav, Suk Wai Lam, Lucile Vanhersecke, Pawel Sobczuk, François Le Loarer, Małgorzata Lenarcik, Piotr Rutkowski, Joris M. van Sabben, Neeltje Steeghs, Hester H. Van Boven, Isidro Machado, Silvia Bagué, Samuel Navarro, Emilio Medina-Ceballos, Carolina Agra-Pujol, Francisco Giner, Gustavo Tapia, Alba Hernández-Gallego, Gema Civantos-Jubera, Miriam Cuatrecasas, Sandra Lopez-Prades, Raul Perret, Isabelle Soubeyran, Emmanuel Khalifa, Laura Blouin, Eva Wardelmann, Alexandra Meurgey, Paola Collini, Artem Voloshin, Yasushi Yatabe, Hidekazu Hirano, Alessandro Gronchi, Toshirou Nishida, Olivier Bouche, Jean-Francois Emile, Carine Ngo, Peter Hohenberger, Cristina Cotarelo, Jens Jakob, Judith V.M.G. Bovee, Hans Gelderblom, Anna Szumera-Ciećkiewicz, Myriam Jean-Denis, Julien Bollard, Nathalie Lassau, Axel Le Cesne, Jean-Yves Blay, Antoine Italiano, Amandine Crombe, Jean-Michel Coindre, Jakob Nikolas Kather

**Affiliations:** 1 Else Kroener Fresenius Center for Digital Health, Faculty of Medicine and University Hospital Carl Gustav Carus, TUD Dresden University of Technology, Dresden, Germany.; 2 Department of Data and Digital Health, Institut Bergonié, Bordeaux, France.; 3 INRIA center at University of Bordeaux, Talence, France.; 4 Bordeaux Mathematics Institute (IMB), CNRS UMR 5251, University of Bordeaux, Talence, France.; 5 Department of Pathology, Leiden University Medical Center, Leiden, the Netherlands.; 6 Department of Biopathology, Institut Bergonié, Bordeaux, France.; 7 Department of Soft Tissue/Bone Sarcoma and Melanoma, Maria Sklodowska-Curie National Research Institute of Oncology, Warsaw, Poland.; 8 Institute of Oncology, BRIC U1312, INSERM, Université de Bordeaux, Institut Bergonié, Bordeaux, France.; 9 Department of Gastroenterology, Hepatology and Clinical Oncology, Centre of Postgraduate Medical Education, Warsaw, Poland.; 10 Department of Cancer Pathology, Maria Sklodowska-Curie National Research Institute of Oncology, Warsaw, Poland.; 11 Department of Medical Oncology, The Netherlands Cancer Institute, Amsterdam, the Netherlands.; 12 Department of Medical Oncology, University Medical Centre Utrecht, Utrecht University, Utrecht, the Netherlands.; 13 Department of Pathology, The Netherlands Cancer Institute, Amsterdam, the Netherlands.; 14 Department of Pathology, Fundación Instituto Valenciano de Oncología, Valencia, Spain.; 15 Patologika Laboratory Hospital Quirón-Salud, Valencia, Spain.; 16 Department of Pathology, University of Valencia, Valencia, Spain.; 17 CIBERONC(ISCIII), Madrid, Spain.; 18 Department of Pathology, Hospital de la Santa Creu i Sant Pau, Barcelona, Spain.; 19 INCLIVA, Valencia, Spain.; 20 Pathology Department, HGU Gregorio Marañon, Madrid, Spain.; 21 Department of Pathology, Hospital Universitari i Politècnic La Fe, Valencia, Spain.; 22 Department of Pathology, Hospital Universitari Germans Trias i Pujol, IGTP, Universitat Autònoma de Barcelona, Barcelona, Spain.; 23 Department of Pathology, Hospital Universitario Virgen del Rocío, Sevilla, Spain.; 24 Pathology Department, Hospital Clinic, Barcelona, Spain.; 25 Liver and Digestive Diseases Biomedical Research Center Network (CIBERehd), Madrid, Spain.; 26 Institut d’Investigacions Biomediques August Pi i Sunyer (IDIBAPS), Barcelona, Spain.; 27 University of Barcelona, Faculty of Medicine, Barcelona, Spain.; 28 Gerhard-Domagk-Institute of Pathology, University Hospital Münster, Münster, Germany.; 29 Biopathology Department, Centre Léon Bérard, Unicancer, Lyon, France.; 30 Department of Advanced Diagnostics, Soft Tissue Tumor Pathology Unit, Fondazione IRCCS Istituto Nazionale dei Tumori di Milano, Milan, Italy.; 31 Department of Diagnostic Pathology, National Cancer Center Hospital, Tokyo, Japan.; 32 Department of Gastrointestinal Medical Oncology, National Cancer Center Hospital, Tokyo, Japan.; 33 Department of Surgery, Fondazione IRCCS Istituto Nazionale dei Tumori, University of Milan, Milan, Italy.; 34 Department of Surgery, Japan Community Healthcare Organization Osaka Hospital, Osaka, Japan.; 35 Department of Surgery, National Cancer Center Hospital, Tokyo, Japan.; 36 Gastrointestinal Oncology Department, CHU Reims, Reims, France.; 37 Paris-Saclay University, Versailles SQY University, INSERM U981, Assistance Publique–Hôpitaux de Paris (AP-HP), Ambroise-Paré Hospital, Smart Imaging, Service de Pathologie, Boulogne, France.; 38 Department of Pathology, Institut Gustave Roussy, Villejuif, France.; 39 Division of Surgical Oncology, Mannheim University Medical Center, Medical Faculty Mannheim, University of Heidelberg, Mannheim, Germany.; 40 Institute of Pathology, Medical Faculty Mannheim, Heidelberg University, Mannheim, Germany.; 41 Department of Surgery, Sarcoma Unit, University Medical Center and Medical Faculty Mannheim, Heidelberg University, Mannheim, Germany.; 42 Department of Medical Oncology, Leiden University Medical Center, Leiden, the Netherlands.; 43 Biobank, Maria Sklodowska-Curie National Research Institute of Oncology, Warsaw, Poland.; 44 Research and Innovation Department, Centre Léon Bérard, Unicancer, Lyon, France.; 45 Imaging Department, Institut Gustave Roussy, BIOMAPS, UMR1281, University Paris-Saclay, Villejuif, France.; 46 Department of Medical Oncology, Institut Gustave Roussy, Villejuif, France.; 47 Department of Medicine, Centre Leon Berard, University Claude Bernard Lyon, Villeurbanne, France.; 48 Department of Medicine, Institut Bergonié, Bordeaux, France.; 49 Department of Oncologic Imaging, Institut Gustave Roussy, Villejuif, France.; 50 Department of Medicine I, Faculty of Medicine and University Hospital Carl Gustav Carus, TUD Dresden University of Technology, Dresden, Germany.; 51 Medical Oncology, National Center for Tumor Diseases (NCT), University Hospital Heidelberg, Heidelberg, Germany.; 52 Pathology and Data Analytics, Leeds Institute of Medical Research, St James’s University of Leeds, Leeds, United Kingdom.

## Abstract

Gastrointestinal stromal tumor (GIST) is the most common gastrointestinal mesenchymal tumor, driven by tyrosine–protein kinase (*KIT*) and platelet-derived growth factor receptor A (*PDGFRA*) mutations. Specific variants, such as *KIT* exon 11 deletions, carry prognostic and therapeutic implications, whereas wild-type variants derive limited benefit from tyrosine kinase inhibitors. Given the limited reproducibility of established clinicopathologic risk models, deep learning (DL) applied to whole-slide images (WSI) emerged as a promising tool for molecular classification and prognostic assessment. We analyzed 8398 GIST cases from 21 centers in seven countries, including 7,238 with molecular data and 2,638 with clinical follow-up. DL models were trained on WSIs to predict mutations, treatment sensitivity, and recurrence-free survival (RFS). DL predicted mutational status in GIST from WSIs, with area under the curve of 0.87 for *KIT* and 0.96 for *PDGFRA*, and high performance was observed for subtypes, including *KIT* exon 11 del–inss 557 to 558 (0.67) and *PDGFRA* exon 18 D842V (0.93). For therapeutic categories, performance reached 0.84 for avapritinib sensitivity and 0.81 for imatinib sensitivity. DL models predicted RFS, with hazard ratios of 8.44 in the overall cohort and 4.74 in patients receiving adjuvant therapy. Prognostic performance was comparable with pathology-based scores, with highest discrimination in the overall cohort and in patients without adjuvant therapy. DL applied to WSIs enables prediction of molecular alterations, treatment sensitivity, and RFS in GIST, performing comparably with established risk scores across international cohorts, providing a baseline for future multimodal predictors.

**Significance:**

Deep learning on histology predicts *KIT* and *PDGFRA* mutations and stratifies recurrence-free survival in a large international cohort of gastrointestinal stromal tumors from multiple centers.

## Introduction

Gastrointestinal stromal tumors (GIST) represent the most common mesenchymal neoplasms of the gastrointestinal tract (1), with an annual incidence of 10 to 15 per million (2, 3) and a median age at diagnosis of approximately 60 to 65 years. Most originate in the stomach and small intestine (4), and less commonly, they arise in the duodenum, rectum, colon, or esophagus (5).

Molecularly, most GISTs harbor activating mutations in tyrosine–protein kinase [*KIT* (60%–70%)] or platelet-derived growth factor receptor A [*PDGFRA* 10% to 15% (6, 7)], both of which carry important prognostic and therapeutic implications. *KIT* exon 11 mutations, particularly at codons 557/558, are linked to aggressive behavior but predict strong benefit from adjuvant imatinib (8, 9). *KIT* exon 9 mutations (6%–9%; ref. 8) typically require higher imatinib dosing for optimal efficacy in advanced phase, though not in the adjuvant setting (10). *PDGFRA* exon 18 D842V mutations confer resistance to most tyrosine kinase inhibitors (TKI) but respond to avapritinib (11–13). Conversely, non-D842V *PDGFRA* exon 18 and exon 12 mutations remain generally imatinib-sensitive (14). Approximately 10% to 15% of GISTs are wild-type (WT) for both genes, including SDH-deficient and NF1-associated subgroups, which derive limited benefit from current TKIs (8, 12). Nevertheless, other mutations exist within WT GISTs, albeit with very low prevalence, such as those occurring in *BRAF* (sensitive to *BRAF* inhibitors) and in neurotrophic tyrosine receptor kinase (*NTRK*) family genes (sensitive to *NTRK* inhibitors; refs. 8, 14).

Risk stratification is currently based on clinicopathologic features such as tumor size, mitotic index, and site, with widely used systems including the NIH consensus criteria, the Armed Forces Institute of Pathology (AFIP, Miettinen–Lasota) classification, and the modified Miettinen–Joensuu (MJ) criteria (e.g., AFIP/MJ criteria; refs. 9, 15). These systems, though widely used, are limited by interobserver variability and reliance on subjective criteria like mitotic counting (16), underscoring the need for more objective prognostic tools (17).

Deep learning (DL) applied to hematoxylin–eosin (H&E) whole-slide images (WSI) for the extraction of prognostic and predictive morphologic features has emerged as a promising approach. Prior studies have demonstrated the capacity of DL to classify prevalent GIST mutations and forecast patient outcomes (11) as well as to predict response to TKIs (12). However, the generalizability of such models across heterogeneous clinical settings remains to be established through evaluation in larger, more diverse cohorts with rigorous external validation (11, 12).

Such approaches should be considered complementary to, rather than replacements for, molecular testing, serving as an efficient prescreening tool to prioritize or rule out patients before molecular analysis.

To address these limitations, we assembled more than 8,000 WSIs from 21 institutions in seven countries from Europe and Asia, with harmonized clinical, pathologic, molecular, and follow-up data. Leveraging state-of-the-art foundation models and transformer architectures, we aimed to (i) predict *KIT* and *PDGFRA* mutation subtypes directly from WSIs, (ii) develop an integrated prognostic model for recurrence-free survival (RFS) incorporating adjuvant imatinib, and (iii) identify subgroups most likely to benefit from targeted therapy.

## Materials and Methods

### Patient data acquisition

We assembled a multicenter international cohort comprising 8,045 WSIs from 21 institutions across seven countries (France, Spain, the Netherlands, Germany, Italy, Poland, and Japan; Supplementary Table S1). This full multicenter study population is referred to throughout the article as the DIGIST cohort. Comprehensive clinical, pathologic, and molecular data were collected for each participant; Supplementary Table S2. Eligibility required a gastrointestinal tumor morphology consistent with GIST and positive immunostaining for *KIT* and/or DOG1. All samples were obtained from tumors that were chemotherapy- and TKI-naïve at the time of tissue collection. Collected variables included sex, age at diagnosis, relevant medical history (NF1, familial GIST, and Carney triad), type of sampling (resection, open biopsy, and microbiopsy), tumor location, size, mitotic count, AFIP risk group, surgery, rupture, adjuvant TKI therapy, recurrence, and follow-up status. All participating centers were specialized in soft tissue tumors, and risk stratification according to the AFIP/MJ criteria was harmonized by an expert pathologist (J.M. Coindre). Molecular data were curated at gene, exon, and codon levels.

This study was conducted in accordance with the Declaration of Helsinki. It involves a retrospective analysis of scanned images derived from anonymized tissue samples of various cancer patient cohorts. Written informed consent was obtained from all individual participants included in the study. All data were subsequently anonymized for this research. The overall retrospective analysis was approved by the Ethics board of the Medical Faculty of Technical University Dresden (Approval # BO-EK-444102022_2).

### Image processing and DL techniques

#### Whole-slide digitization

Slides were stained with H&E in most centers, whereas French centers used H&E–saffron (HES; Supplementary Table S1). WSIs were digitized at 40× magnification using local scanners, including Hamamatsu NanoZoomer, 3dhistech Pannoramic, and Aperio AT2 systems. Slides from both staining protocols were included in the pooled analysis, as H&E and HES are closely related routine brightfield histology stains that preserve the same overall tissue morphology. Nevertheless, cross-stain compatibility was not directly evaluated in the present study and remains a limitation.

#### Data preprocessing—WSI

WSIs were preprocessed with the STAMP pipeline (18) for tessellation and background rejection; patches were saved as JPEGs and subsequently used for feature extraction with CONCH (19) to train the downstream Vision Transformer (ViT) classification models. For each mutation, dichotomous variables were generated (positive for mutated; negative otherwise). Missing molecular tests were left blank per pipeline specifications. CONCH-derived features and the clinical or molecular data served as input for ViT (arxiv 2010.11929) classification model trained utilizing the default parameters as specified in the STAMP pipeline documentation (18).

#### Data preprocessing—clinical data

Clinical and molecular metadata were collected via a standardized template. Substantial intersite heterogeneity in language, date formats, and mutation reporting required harmonization. Molecular information provided as nucleotide or protein formulas was standardized into categorical variables (gene/exon and codon level) using canonical Ensembl transcripts (*KIT*: NM_000222.2; *PDGFRA*: NM_006206.4). Key subtypes, including *KIT* exon 9 and *KIT* exon 11 ≥ 2 codon deletions/del–ins, were automatically identified using the *hgvs.parser* library (20).

To ensure dataset consistency, we developed the Grammar Data Curation Tool (GraDaCu), a Python-based interface enforcing structured schema definitions, mandatory fields, and value constraints Supplementary Table S2. Iterative automated and manual checks ensured conformity before exporting a curated CSV for downstream analysis.

#### Mutation/clinical classification model

For the mutation prediction analysis, we first identified the overarching cohort of GIST samples with available molecular mutational data (*n* = 7,238). To evaluate model generalizability across tissue procurement methods, these cases were subsequently stratified by sample type into surgical resections and biopsies. Model training and internal validation were strictly restricted to the resection samples.

To ensure rigorous external validation, we used a geographic split. Resection samples sourced from France, Germany, the Netherlands, and Japan were partitioned at a ratio of 80:20 into training and internal validation cohorts, utilizing stratified sampling to preserve key clinical variables. Conversely, to prevent geographic data leakage, all samples originating from Spain, Italy, and Poland were entirely withheld from the training phase. From these held-out countries, we constructed two independent external validation cohorts: one composed exclusively of resections and the other composed exclusively of biopsies (*n* = 223). This strict partitioning ensured that the model was externally validated against both an unseen geographic population and an entirely unseen tissue modality.

Mutation prediction models utilized DL frameworks based on STAMP and COBRA (arxiv 2411.13623), with the latter evaluated under three fine-tuning strategies. Mutation prediction via STAMP utilized a ViT network adapted for multiple instance learning. Extracted CONCH tile features were processed through two self-attention transformer layers alongside a classification token to generate a unified slide-level prediction. Models were trained on bags of 512 randomly sampled tiles per slide (batch size = 64) for up to 32 epochs, using a maximum learning rate of 1 × 10^−4^ and early stopping with a patience of 16. Class-weighted loss functions were also applied to correct for underlying mutation prevalence imbalances.

Model performance area under the curve (AUC) was assessed by bootstrap resampling (1,000 iterations) to derive 95% confidence intervals (CI). Models included DL approaches based on STAMP and COBRA, the latter trained under three fine-tuning strategies. For explainability, the most predictive tiles were extracted, and heatmaps were generated to visualize the precise regions of model focus.

#### RFS model

For RFS, the DIGIST cohort was partitioned using the same geographic scheme as the mutation model (C1, all patients; C2, without adjuvant therapy; and C3, with adjuvant therapy) excluding patients with a follow-up shorter than 12 months (Supplementary Fig. S1A).

To predict RFS, we developed an attention-based multiple instance learning model. The architecture extracts CONCH foundation features from individual tissue tiles, utilizing an attention mechanism to weight and aggregate these tiles into a comprehensive slide-level prediction. Training was conducted using bags of 512 randomly sampled tiles per slide, optimizing for 32 epochs with a maximum learning rate of 1 × 10^−4^ and a batch size of 64. A class-weighted cross-entropy loss function was applied to address training imbalances. The final model was selected based on peak validation ROC–AUC, with all available slide tiles utilized during clinical inference.

Different DL models were trained on C1, C2, and C3 training sets to address the prognostic tasks and were subsequently named DL scores C1, C2, and C3. For each cohort, a same pipeline was used to evaluate the prognostic value of the scores and their added value compared with the mitotic count alone and combined with the other established prognostic variables from the AFIP criteria (i.e., tumor size, location, and rupture; Supplementary Fig. S1B). Age, sex, and mutational status (as well as adjuvant TKI therapy for the C1 cohort) were systematically included as baseline covariates in all models to account for their potential confounding effect and to ensure consistent risk adjustment across model comparisons. Overall, five models were systematically trained on training C1, training C2, and training C3 relying on the (i) DL score (named simple DL model); (ii) DL score combined with tumor size, location, and rupture (named deep MJ model); (iii) mitotic count (named mitotic model); (iv) AFIP criteria provided by the pathologists (named pathologic MJ model); and (v) mitotic count combined with tumor size, location, and rupture (named continuous MJ model). The deep MJ model was included as a hypothesis-driven integrative model to assess whether combining the DL score with established MJ variables could improve prognostic performance beyond either modality alone. The performance of these DL-based models were systematically compared against the three other benchmark models providing insights into whether Artificial Intelligence–driven approaches can enhance or complement existing prognostic tools.

Univariable survival analyses including log-rank tests and Cox regressions were first performed in each training cohort on the variables entered in the models.

Afterward, the multivariable models were trained using the Cox proportional hazard ratio (HR) model in the training cohorts and applied on the internal and external validation cohorts. Multivariable HRs with their 95% CIs were calculated in the training cohorts. The performances of the models were estimated using Harrell concordance index (C-index, which assesses the discriminative ability of the models) and integrated Brier score (IBS, combined with prediction error curves, which quantifies overall prediction accuracy and calibration over time) over the first 10 years following resection with 95% CIs. In the training cohorts, the model performances were evaluated using fivefold cross-validation, with evaluation performed on the out-of-fold samples (Supplementary Fig. S1B).

Model performances were then compared in the internal and external validation cohorts using permutation tests, in which predicted risks were randomly swapped (with 1,000 random label exchanges) between models to generate the null distribution of differences in Harrell’s C-index and IBS. *P* values were computed as the proportion of permuted differences exceeding the observed one. Kaplan–Meier curves for RFS in each patient group were drawn using training-set median cut points.

## Results

### DL can predict mutational status in GIST directly from pathology slides

We evaluated whether foundation model–based DL pipelines could predict mutational status directly from H&E WSIs. Using a large multicentric, international cohort of surgically resected GISTs, we trained classifiers for 12 mutation categories and validated them on external cohorts.

**Figure 1.**
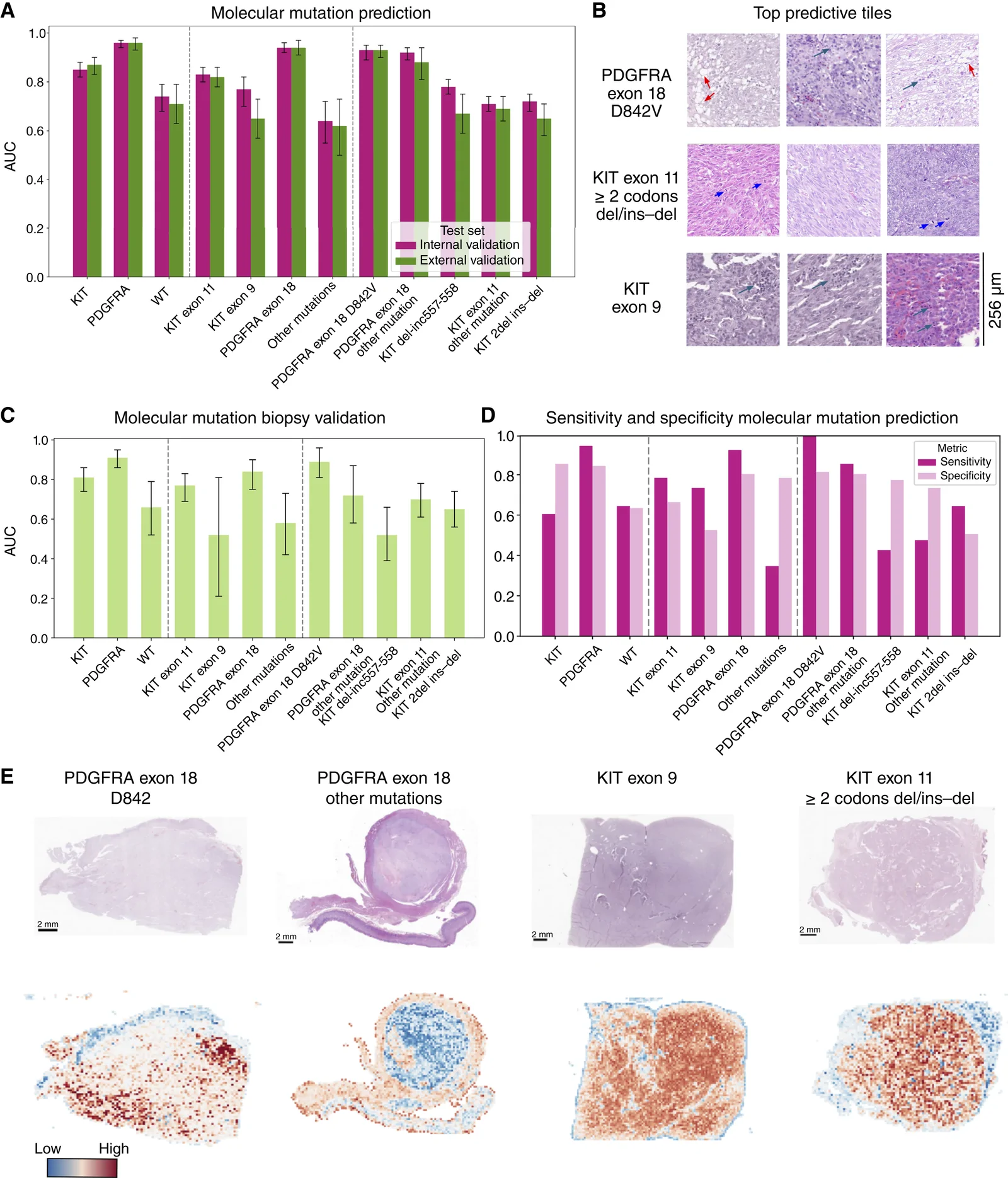
DL accurately predicts key molecular mutations in GIST from histopathology. **A,** AUC with 95% CIs for DL-based prediction of selected molecular mutations in the internal validation cohort (pink) and the external validation cohort (green). **B,** Representative top-ranked predictive image tiles for three different mutations (*PDGFRA* exon 18 D842V, *KIT* exon 11 with two or more codons deleted, and *KIT* exon 9), as identified by the model’s attention score. Green arrows, lymphocytes for *KIT* exon 9 and *PDGFRA* exon 18 D842V; red arrows, vacuolization of cells for *PDGFRA* exon 18 D842V; blue arrows, mitoses for KIT exon 11 ≥ 2 codons/del–ins. **C,** AUC with 95% CIs for the same model shown in **A** when deployed on an external validation cohort consisting exclusively of biopsy specimens; results are reported for all selected mutations. **D,** Bar plot detailing the sensitivity (pink) and specificity (light pink) for each mutation. **E,** Heatmaps generated via gradient-weighted class activation mapping (Grad-CAM) illustrate the predictive importance of individual tissue tiles for each mutation: red, high prediction score; blue, low prediction score. In the case of well-characterized mutations such as *PDGFRA* exon 18 D842V and *KIT* exon 9, the model focused on tumor regions, in line with reported morphologic correlates. In other cases (*PDGFRA* exon 18 other mutations), mucosal regions were also emphasized, likely due to vacuolated cells resembling *PDGFRA*-mutated morphology. Even for more specific variants, such as *KIT* exon 11 deletions or insertions, the model continued to highlight relevant tumor areas.

DL models accurately predicted *KIT* and *PDGFRA* mutations, with AUCs ranging from 0.87 (95% CI, 0.83–0.90) for *KIT* to 0.96 (95% CI, 0.93–0.98) for *PDGFRA* in external validation (Table 1; Fig. 1A). Performance was particularly strong for *PDGFRA* exon 18, including the clinically relevant D842V variant [AUC 0.93 (95% CI, 0.90–0.95) external], and for *KIT* exon 11 [AUC 0.82 (95% CI, 0.78–0.86)]. By contrast, *KIT* exon 9, WT, and other rare mutations showed only moderate discrimination. To provide interpretability for these classifications, we extracted the highest-ranked image tiles based on the network’s attention scores (Fig. 1B). This tile-level analysis confirmed that the model learned to leverage distinct, variant-specific morphologic hallmarks, such as cellular vacuolization, varying mitotic activity, and localized immune cell infiltrates, to differentiate between key *PDGFRA* and *KIT* mutational subtypes.

**Table 1.** Predictive performance of the DL models for selected molecular mutations in GIST.

Molecular mutation	Test set	AUC	CI lower	CI upper
KIT	Internal test set	0.85	0.82	0.88
KIT	External validation	0.87	0.83	0.9
KIT	External validation biopsy only	0.81	0.74	0.86
PDGFRA	Internal test set	0.96	0.94	0.97
PDGFRA	External validation	0.96	0.93	0.98
PDGFRA	External validation biopsy only	0.91	0.86	0.95
WT	Internal test set	0.74	0.68	0.79
WT	External validation	0.71	0.63	0.79
WT	External validation biopsy only	0.66	0.52	0.79
KIT exon 11	Internal test set	0.83	0.8	0.86
KIT exon 11	External validation	0.82	0.78	0.86
KIT exon 11	External validation biopsy only	0.77	0.69	0.83
KIT exon 9	Internal test set	0.77	0.7	0.82
KIT exon 9	External validation	0.65	0.57	0.73
KIT exon 9	External validation biopsy only	0.52	0.21	0.81
PDGFRA exon 18	Internal test set	0.94	0.92	0.96
PDGFRA exon 18	External validation	0.94	0.91	0.97
PDGFRA exon 18	External validation biopsy only	0.84	0.75	0.9
Other mutations	Internal test set	0.64	0.55	0.72
Other mutations	External validation	0.62	0.5	0.73
Other mutations	External validation biopsy only	0.58	0.42	0.73
PDGFRA exon 18 D842V	Internal test set	0.93	0.89	0.95
PDGFRA exon 18 D842V	External validation	0.93	0.9	0.95
PDGFRA exon 18 D842V	External validation biopsy only	0.89	0.81	0.96
PDGFRA exon 18 other mutation	Internal test set	0.92	0.89	0.94
PDGFRA exon 18 other mutation	External validation	0.88	0.81	0.94
PDGFRA exon 18 other mutation	External validation biopsy only	0.72	0.58	0.87
KIT del-inc557-558	Internal test set	0.78	0.75	0.81
KIT del-inc557-558	External validation	0.67	0.59	0.75
KIT del-inc557-558	External validation biopsy only	0.52	0.39	0.66
KIT exon 11 other mutation	Internal test set	0.71	0.68	0.74
KIT exon 11 other mutation	External validation	0.69	0.64	0.74
KIT exon 11 other mutation	External validation biopsy only	0.7	0.61	0.78
KIT 2del ins–del	Internal test set	0.72	0.68	0.75
KIT 2del ins–del	External validation	0.65	0.58	0.71
KIT 2del ins–del	External validation biopsy only	0.65	0.56	0.74

NOTE: Results are reported as the AUC with 95% CI. For each predicted mutation, the AUC column details model performance across the internal test set, the independent external validation set, and the biopsy-only external validation subset. The WT group is defined by tumors with no mutations in both KIT and PDGFRA. The other mutation group includes all mutations that are not categorized as KIT exon 11, KIT exon 9, PDGFRA exon 18, or WT.

In a biopsy-only external validation cohort (*n* = 223), performance declined, consistent with the smaller tissue area. *KIT* and *PDGFRA* remained well predicted with AUCs 0.81 (95% CI, 0.74–0.86) and 0.91 (95% CI, 0.86–0.95), respectively, whereas WT and rare variants were modest (AUCs ≤0.70; Fig. 1C; Table 1).

External validation confirmed the robustness of the models (Fig. 1D). *KIT* predictions reached sensitivity 0.61 and specificity 0.86, whereas *PDGFRA* achieved sensitivity 0.95 and specificity 0.85. The *PDGFRA* D842V variant reached perfect sensitivity (1) with specificity 0.82. Overall *KIT* exon 11 and *PDGFRA* predictions achieved the highest F1 scores (0.75–0.83). Full subgroup results are provided in Table 2.

**Table 2.** Sensitivity, specificity, and F1-score for molecular mutation prediction.

Molecular mutation	Sensitivity	Specificity	F1_opt	F1_sens90
KIT	0.61	0.86	0.83	0.84
PDGFRA	0.95	0.85	0.78	0.29
WT	0.65	0.64	0.31	0.19
KIT exon 11	0.79	0.67	0.82	0.77
KIT exon 9	0.74	0.53	0.19	0.13
PDGFRA exon 18	0.93	0.81	0.75	0.26
Other mutations	0.35	0.79	0.15	0.10
KIT del-inc557-558	0.43	0.78	0.33	0.23
KIT exon 11 other mutation	0.48	0.74	0.66	0.59
PDGFRA exon 18 D842V	1.00	0.82	0.49	0.14
PDGFRA exon 18 other	0.86	0.81	0.75	0.26
KIT 2del ins–del	0.65	0.51	0.42	0.36

NOTE: Performance metrics of the DL model are reported for all molecular mutation categories in the external validation cohort. Values are presented for the optimal classification threshold determined by Youden’s index and for a fixed sensitivity of 90%.

Using a high-sensitivity threshold is appropriate for clinical prescreening before confirmatory gold-standard testing. At 90% sensitivity, the number needed to test was 1.15 for *KIT*, 1.45 for *PDGFRA*, and 7.0 for WT, corresponding to a relative reduction of 5%, 88%, and 30%, respectively.

In line with recent advances in DL-based molecular prediction, we also evaluated the COBRA workflow and observed results comparable with our baseline foundation model (Supplementary Table S3).

We conducted an explainability analysis to identify histopathologic features associated with specific mutations and clinical outcomes. Heatmaps generated using gradient-weighted class activation mapping (arxiv 1610.02391; Fig. 1E) highlighted the regions most relevant for prediction. These heatmaps and the corresponding top-ranked predictive tiles were qualitatively reviewed by an expert pathologist to assess whether the model was focusing on biologically meaningful tumor regions rather than potentially confounding contextual features. When examining these regions and the most predictive tiles for different mutations, we observed that the *PDGFRA* exon 18 D842V [Fig. 1E (left)] mutation was associated with epithelioid cell morphology, cytoplasmic vacuolization, myxoid stroma, and lymphoid infiltrates. In contrast, *KIT* exon 11 [Fig. 1E (right)] mutations involving deletions of two or more codons were linked to mitotic activity and hyperchromasia, whereas *KIT* exon 9 mutations were associated with lymphoid infiltrates.

### DL predicts clinically actionable alterations from pathology slides

**Figure 2.**
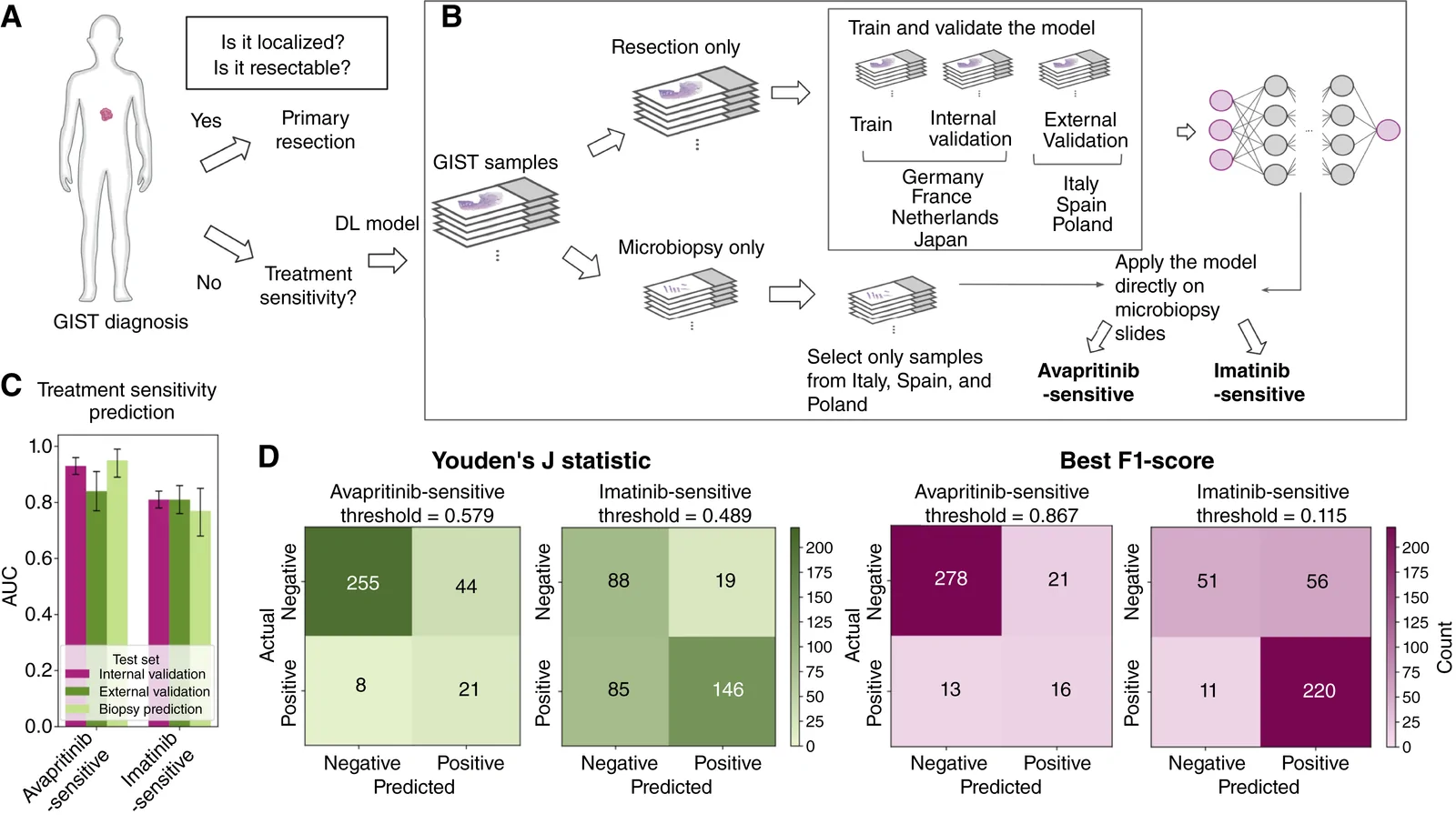
DL predicts treatment sensitivity and supports clinical decision-making in GIST. **A,** Standard clinical workflow from initial diagnosis to treatment selection in patients with GIST. **B,** Proposed integration of the DL model into the diagnostic–therapeutic pathway, enabling early triage of patients for further molecular analysis or tailored treatment selection. **C,** AUC with 95% CI for DL-based prediction of treatment sensitivity, reported for the internal validation cohort (pink), external validation cohort (green), and an external biopsy-only validation cohort (light green). **D,** Confusion matrices for avapritinib and imatinib sensitivity, shown at the optimal threshold by Youden’s index (green) and at the threshold yielding the highest F1 score (pink).

We grouped mutations into three actionable categories: avapritinib-sensitive, imatinib-sensitive, and imatinib dose adjustment as defined in Kong and colleagues (Supplementary Table S4; ref. 12) and trained DL models to predict each category from H&E slides (Fig. 2A and B).

DL achieved strong predictive performance for avapritinib sensitivity [AUC 0.84 (95% CI, 0.77–0.91)] and consistent accuracy for imatinib sensitivity [AUC 0.81 (95% CI, 0.76–0.86)]. By contrast, predictions for imatinib dose adjustment were weaker (AUCs ≤0.73; Table 3; Fig. 2C).

**Table 3.** Predictive performance of the DL models for treatment-sensitivity categories in GIST.

Treatment	Test set	AUC	CI lower	CI upper
Avapritinib-sensitive	Internal test set	0.93	0.9	0.96
Avapritinib-sensitive	External validation	0.84	0.77	0.91
Avapritinib-sensitive	External validation biopsy only	0.95	0.89	0.99
Imatinib-sensitive	Internal test set	0.81	0.78	0.84
Imatinib-sensitive	External validation	0.81	0.76	0.86
Imatinib-sensitive	External validation biopsy only	0.77	0.68	0.85
Imatinib dose adjustment	Internal test set	0.78	0.71	0.84
Imatinib dose adjustment	External validation	0.73	0.64	0.82
Imatinib dose adjustment	External validation biopsy only	0.34	0.04	0.96

NOTE: Results are reported as AUC with 95% CI. For each treatment-sensitivity category, the AUC column details model performance across the internal validation set, the independent external validation set, and the biopsy-only external validation subset.

External validation confirmed predictive capacity, with avapritinib and imatinib sensitivities showing the most robust sensitivity/specificity balance, whereas dose adjustment remained weaker (Table 4; Fig. 2D). The “rule-out fraction” represents the proportion of patients who can be excluded from further testing at a given high-sensitivity threshold. At 90% sensitivity, the rule-out fractions were 0.50 for avapritinib-sensitive, 0.56 for imatinib dose adjustment, and 0.24 for imatinib-sensitive for resections (Table 4).

**Table 4.** Sensitivity and specificity for treatment-sensitivity prediction.

Treatment	Sensitivity	Specificity	F1_opt	F1_sens90	ROF_90%_sens
Avapritinib-sensitive	0.72	0.83	0.45	0.16	0.502
Imatinib dose adjustment	0.65	0.65	0.22	0.11	0.555
Imatinib-sensitive	0.62	0.82	0.74	0.81	0.236

NOTE: DL model performance in the external validation cohort for avapritinib-sensitive, imatinib dose adjustment, and imatinib-sensitive categories.

### DL predicts clinical outcomes in resected GIST

Among the 1,715 patients with available mutation data, genotypes were grouped for analytical clarity into unfavorable (*KIT* exon 11 ≥2 codon deletions/del–ins, *KIT* exon 9) and favorable (WT, *PDGFRA* exon 18) prognostic categories. Patients harboring unfavorable mutations exhibited a significantly elevated risk of disease recurrence (HR 2.74; 95% CI, 2.19–3.42; *P* < 0.0001; Supplementary Table S5).

Given the established impact of adjuvant therapy on GIST prognosis (2), we evaluated the entire cohort of samples with available follow-up data and then performed identical parallel analyses stratified by treatment status (adjuvant TKI vs. no adjuvant TKI). Accordingly, we tested whether DL could directly predict RFS from H&E WSIs in cohort C1 (Supplementary Table S6).

**Figure 3.**
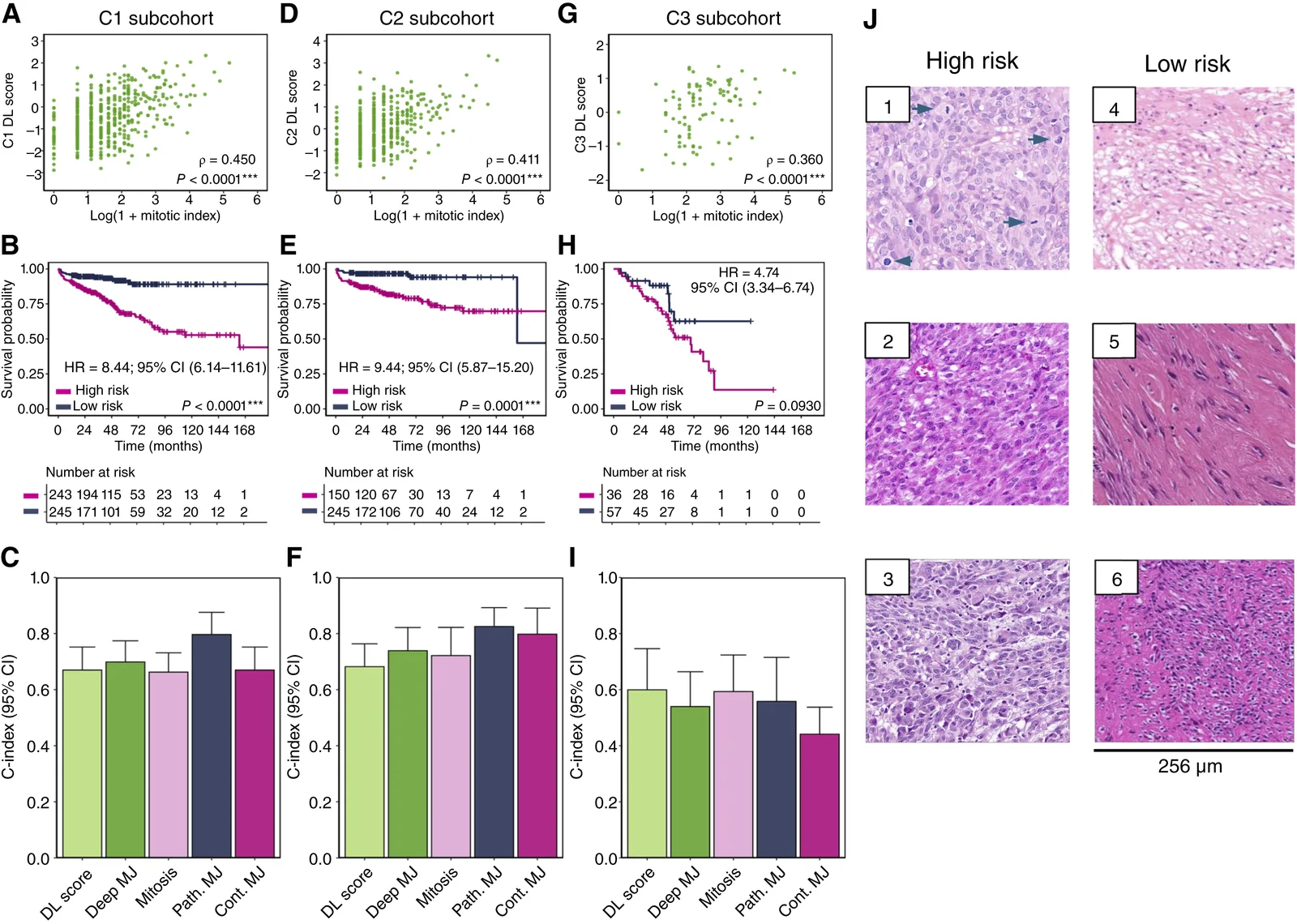
DL stratifies recurrence risk and complements clinical prognostic models in GIST. **A–C,** Analyses in the overall C1 cohort: **A,** correlations between the DL score and the mitotic index assessed by the Spearman rank test; **B,** Kaplan–Meier curves for RFS according to the DL score (binarized at the median value of the C1 training dataset). **C,** C-indices with 95% CIs for five prognostic models: mitotic model (“mitosis”), simple DL model (“DL score”), pathologic MJ model (“Path. MJ”), continuous MJ model (“Cont. MJ”), and deep MJ model (“Deep MJ”). **D–F,** Corresponding analyses in the C2 subcohort (patients from C1 who did not receive adjuvant TKI therapy). **G–I,** Corresponding analyses in the C3 subcohort (patients from C1 who received adjuvant TKI therapy). **J,** Top representative tiles. Tiles 1, 2, and 3 from high-risk tumors showed high cellularity with epithelioid cells, nuclear atypia, and hyperchromasia. Tile 1 showed numerous mitoses (arrows). Tiles 4, 5, and 6 from low-risk tumors showed low cellularity with spindle cells and fibrosis, no atypia and no hyperchromasia. ***, *P* < 0.001.

The DL score strongly correlated with the mitotic count (*P* < 0.0001; Fig. 3A) and provided significant prognostic information. When dichotomized at the training-set median, the DL score stratified patients into high- and low-risk groups with highly significant differences in RFS [HR of 8.44; 95% CI, 6.14–11.61; *P* < 0.0001; Fig. 3B Supplementary Table S5]. In external validation, the simple DL model achieved a C-index of 0.67 (95% CI, 0.59–0.75), comparable with the mitotic index (0.66, 95% CI, 0.60–0.73) but lower than the AFIP criteria (0.80, 95% CI, 0.74–0.88; Fig. 3C; Table 5; full results in Supplementary Table S7).

**Table 5.** Prognostic performance of mitotic index, DL score, and MJ risk models in cohorts C1 to C3.

Cohort	Model	C-index (95% CI)	HR high vs. low DL (95% CI)	*P* value
C1	Mitotic model	0.66 (0.60–0.73)	—	—
	Simple DL model	0.67 (0.59–0.75)	8.44 (6.14–11.61)	<0.0001
	Pathologic MJ model	**0.80 (0.74–0.88)**	—	—
	Deep MJ model	0.70 (0.63–0.77)	—	—
C2	Mitotic model	0.72 (0.64–0.82)	—	—
	Simple DL model	0.68 (0.59–0.76)	9.44 (5.87–15.20)	<0.0001
	Pathologic MJ model	**0.83 (0.76–0.89)**	—	—
	Deep MJ model	0.74 (0.64–0.82)	—	—
C3	Mitotic model	0.59 (0.46–0.72)	—	—
	Simple DL model	**0.60 (0.44–0.75)**	4.74 (3.34–6.74)	<0.0001
	Pathologic MJ model	0.56 (0.41–0.71)	—	—
	Deep MJ model	0.54 (0.38–0.66)	—	—

NOTE: C-index values (95% CI) are reported for each model. For the simple DL model, HRs for high versus low DL score (binarized at the median in the C1 training set) with 95% CIs and corresponding *P* values from univariable Cox regression are shown. Cohort C1 includes all patients, C2 comprises patients without adjuvant TKI therapy, and C3 those with adjuvant TKI therapy. Bold values indicate the best-performing model.

In multivariable Cox analyses, the continuous DL score remained a strong independent predictor of RFS (HR 2.83, 95% CI, 2.47–3.25; *P* < 0.0001; Supplementary Table S8), whereas the mitotic index did not (*P* = 0.175). This demonstrates that the DL score provides independent prognostic information beyond established histopathologic criteria. We also evaluated a hypothesis-driven integrated model combining the DL score with MJ clinical variables (deep MJ) to test whether joint modeling could improve prognostic discrimination. This approach modestly improved external discrimination relative to the simple DL model (C-index 0.70; 95% CI, 0.63–0.77; Table 5; full results in Supplementary Table S7) but did not consistently outperform the pathologic MJ model alone, which retained the best external performance. Overall, the DL score correlated with mitotic activity, stratified risk across molecular subtypes, and remained an independent predictor of prognosis, though its external performance was less robust than that of the AFIP score, likely reflecting cohort-level heterogeneity.

### DL retains prognostic value across treatment subgroups

We next evaluated prognostic performance in patients without adjuvant TKI (C2; Supplementary Table S9) and in those receiving adjuvant TKI (C3; Supplementary Table S10). In both cohorts, the DL score correlated strongly with mitotic count (*P* < 0.0001; Fig. 3D), supporting its biological relevance.

In C2, results mirrored those of the overall cohort C1. Kaplan–Meier curves showed clear separation between DL high- and low-risk groups (log-rank *P* < 0.0001; Fig. 3E), with Cox regression confirming a strong prognostic effect (HR 9.44, 95% CI, 5.87–15.20; *P* < 0.0001; Supplementary Table S5). The DL model achieved a C-index of 0.68 (95% CI, 0.59–0.76) in external validation, modestly lower than the mitotic index (0.72, 95% CI, 0.64–0.82) and deep MJ (0.74, 95% CI, 0.64–0.82; Fig. 3F; Table 5; full results in Supplementary Table S7).

In C3, the DL score correlated strongly with mitotic count (*P* < 0.0001; Fig. 3G). In Kaplan–Meier analysis, the survival difference between DL high- and low-risk groups did not reach statistical significance (log-rank *P* = 0.093; Fig. 3H). However, Cox regression still demonstrated a strong prognostic effect (HR 4.74, 95% CI, 3.34–6.74; *P* < 0.0001; Supplementary Table S5). Adding DL to AFIP did not further improve performance, despite the multivariable cox regression demonstrating a strong, independent prognostic effect. The DL model achieved a C-index of 0.60 (95% CI, 0.44–0.75) comparable with the mitotic index (0.59, 95% CI, 0.46–0.72) and superior to AFIP (0.56, 95% CI, 0.41–0.71; Fig. 3I; Table 5, Supplementary Table S7).

When focusing on patients with imatinib-sensitive mutations, the DL score provided particularly sharp stratification for both RFS and OS. Among patients with a high AFIP score but not receiving adjuvant TKIs, DL low-risk cases had significantly better RFS and OS (log-rank *P* = 0.0007 and *P* = 0.0333; Supplementary Fig. S2A and S2B; Supplementary Table S11). Conversely, among patients with high AFIP score receiving adjuvant TKIs, the DL score separated individuals into clinically meaningful subgroups for both RFS and OS (log-rank *P* < 0.0001 for both; Supplementary Fig. S2B–S2D; Supplementary Table S11).

Comparisons across treatment arms further underscored this effect. DL low-risk patients without adjuvant TKIs had higher recurrence and worse OS compared with those receiving therapy, suggesting that a time-limited treatment (∼36 months) may be sufficient. By contrast, DL high-risk patients had poor outcomes regardless of treatment, with median recurrence time (∼60 months) exceeding the median treatment duration (∼35 months), supporting the potential need for prolonged or lifelong TKI therapy (Supplementary Fig. S2; Supplementary Table S11). Representative predictive tiles are shown in Fig. 3J.

Overall, the DL score retained prognostic value across treatment subgroups. Although discrimination was modestly lower than some traditional criteria and Kaplan–Meier separation was weaker in C3, Cox regression consistently confirmed significance. Importantly, subgroup analyses suggest that the DL score could help guide treatment duration by identifying low-risk patients for time-limited therapy and high-risk patients who may require prolonged or lifelong treatment, consistently with a recently published study showing longer duration benefit in high-risk patients (21).

## Discussion

We conducted a comprehensive, multicenter international analysis to predict molecular status and stratify recurrence risk in GISTs directly from routinely available pathology slides. Our findings demonstrate that DL models, trained on a large and diverse cohort of more than 8,000 samples from 21 institutions across seven countries, can accurately predict the most common and clinically actionable *KIT* and *PDGFRA* mutation subtypes directly from native tumor morphology. Crucially, this study validates that a DL-derived score provides strong, independent prognostic information for RFS. This work addresses a critical limitation of previous studies (11) like small cohort size and limited generalizability by confirming the robustness of DL across varied international cohorts and demonstrating its utility as a noninvasive, complementary prescreening tool to augment traditional GIST risk stratification and potentially guide adjuvant therapy duration.

As detailed by the European Society For Medical Oncology Guidelines Committee (2), the molecular characterization of *KIT* and *PDGFRA* mutations is widely recognized as an essential component of the standard diagnostic step for all GISTs. Beyond confirming the diagnosis in morphologically or immunohistochemically ambiguous cases (such as rare CD117/DOG1-negative tumors), mutational profiling provides critical prognostic insight and serves as the primary predictive biomarker for sensitivity to targeted therapies. Although traditional sequencing remains the gold standard for this profiling, there is a well-established correlation between the GIST mutational landscape and distinct histologic features (22, 23). Because the diagnosis of GIST relies heavily on morphology and immunohistochemistry, pathology slides are routinely generated during the standard diagnostic workflow. This creates a compelling opportunity to extract molecular data directly from these standard slides (11). Such an approach not only expedites the determination of mutational status but also optimizes clinical resource allocation by restricting conventional molecular testing to diagnostically ambiguous cases.

Leveraging this biological link to address a fundamental clinical need, our DL models for molecular prediction achieved high accuracy for *KIT* and *PDGFRA* mutations, including near-perfect performance for *PDGFRA* exon 18 D842V (genotype conferring primary resistance to imatinib; refs. 2, 8) and strong discrimination of *KIT* exon 11 variants. By contrast, *KIT* exon 9, WT, and rare subtypes were predicted with only moderate accuracy, reflecting both biological heterogeneity and smaller sample sizes. Importantly, DL outperformed models trained solely on clinical variables, underscoring that morphologic features capture rich genetic information. The robustness of DL predictions across both resections and biopsies supports their potential clinical utility, although performance on biopsy specimens was slightly lower, consistent with the reduced amount of tissue available for analysis. The clinical relevance of molecular prediction is particularly significant for populations facing economic constraints, such as those in low- and middle-income countries, where resource-intensive sequencing diagnostics are often unavailable or prohibitively expensive as highlighted in (24). The DL approach provides a cost-effective triage strategy to streamline case selection for confirmatory molecular testing.

To streamline clinical implementation, we explored a generalized approach grouping distinct mutations into broader treatment-sensitivity categories to serve as a proof of concept for models that directly predict therapeutic outcomes rather than individual molecular variants. When grouped into clinically actionable categories, DL predictions distinguished avapritinib- and imatinib-sensitive tumors with high accuracy, whereas dose adjustment was less reliable. However, the overall performance of these grouped treatment-sensitivity models was inferior to that of the mutation-specific models, particularly in the biopsy-only external validation cohort. This attenuation in performance is likely attributable to the underlying morphologic heterogeneity of the diverse mutations amalgamated within each treatment group. Nevertheless, although the ability to compute “rule-out fractions” at high sensitivity suggests that these generalized models could serve as rapid prescreening tools in resource-limited settings, the mutation-specific approach currently remains the superior strategy for the precise prediction of treatment sensitivity.

For outcome prediction, the DL score correlated strongly with mitotic activity and stratified patients into distinct risk groups. In the overall cohort, DL achieved C-indices similar to the mitotic index but lower than AFIP but remained independently prognostic in multivariable models. Notably, the mitotic index lost significance when modeled alongside DL, indicating that DL captures its prognostic signal more reproducibly. Mutation grouping into unfavorable versus favorable provided a clinically meaningful simplification, consistent with prior reports (25) and illustrates how DL-derived scores can be integrated with molecular features.

Stratification by treatment status provided further insights. In patients without adjuvant TKI therapy, DL-based discrimination was modestly lower than that of classical criteria but still enabled clear risk separation. In patients receiving adjuvant therapy, DL performed comparably with the mitotic index and better than AFIP, although Kaplan–Meier separation was weaker in external validation; nevertheless, Cox regression confirmed that the DL score retained significance. Adjuvant imatinib is known to improve RFS in high-risk GIST, yet only one trial has demonstrated an overall survival benefit, favoring 3 years of treatment over 1 year (9). Therefore, the specific patient subgroup deriving the greatest benefit from adjuvant imatinib therapy is still uncertain, as is the optimal duration of treatment. In our study, we analyzed 628 high-risk patients according to the MJ classification and with a mutation sensitive to imatinib. In this group of patients, 445 received adjuvant imatinib and 183 did not. The DL MJ model stratified both patient subgroups into low- and high-risk categories, with the DL low-risk group showing significantly better RFS and overall survival than the DL high-risk group, both in patients without adjuvant imatinib and in those receiving adjuvant imatinib. Moreover, these exploratory findings generate a clinical hypothesis regarding adjuvant therapy duration. The data hint that a standard, time-limited treatment course (36 months) may be adequate for DL low-risk patients, whereas those classified as DL high-risk could potentially benefit from extended therapeutic strategies. However, these observations are strictly hypothesis-generating and require prospective validation.

Finally, explainability analyses conducted with top predictive tiles and heatmaps revealed that DL models focused on tumor regions and morphologic features consistent with established pathologic correlates, as also observed by Fu and colleagues (11). High cellular density and mitotic activity characterized high-risk tumors, whereas *PDGFRA* D842V mutations displayed epithelioid or mixed morphology with cytoplasmic vacuolization, myxoid stroma, and lymphoid infiltration; *KIT* 557/558 deletions showed mitotic activity. These findings demonstrate that DL accurately captures morphologic hallmarks recognized by pathologists, enhancing interpretability and clinical relevance.

Several limitations should be acknowledged. Despite the large dataset, rare genotypes such as *KIT* exon 9, SDH-deficient, or NF1 GISTs remain underrepresented, limiting performance estimates. Future studies should enrich these cohorts and validate generalizability prospectively. Moreover, epidemiologic and pathologic variations in GIST across populations suggest that our dataset may not fully represent the spectrum of Asian disease patterns, as Asian cases were underrepresented in this study (475 samples) compared with the predominantly European cohort. Furthermore, due to the complexity of the task, our current methodology relies on independent models for each class. In the validation cohorts, fewer than 1% of samples yielded high DL scores for both *KIT* and *PDGFRA* models, indicating that ambiguous dual-positive predictions were rare in practice. Future work will aim to develop a broader, multi-target DL framework for the simultaneous prediction of numerous genetic alterations, similar to our recent approach in (26). Finally, our models relied on histopathology but integration with radiology, genomic, and clinical data through multimodal architectures may enhance performance.

In conclusion, DL applied to WSIs accurately predicts molecular alterations, treatment sensitivity, and recurrence risk in GIST. Although AFIP remains a strong comparator, DL provides independent prognostic information, identifies clinically actionable alterations, and refines treatment stratification. By combining predictive accuracy with interpretability, DL has the potential to complement molecular testing, guide adjuvant therapy decisions, and support more personalized management of GIST.

## Supplementary Material

Figure S1 Statistical pipeline for recurrence-free survival (RFS) analysis. (A) Data partitioning. Boxes with light dashed contours corresponded to the patients with available DL Scores while boxes with solid thick contours to the patient included in the survival analysis. (B) Methodological approach performed in C1, C2 and C3 to obtain deep learning (DL) scores and to develop survival models in the Training cohorts using the Cox proportional hazard (CPH) algorithm and to compare them. Other abbreviations: ext.val: external validation, int. val: internal validation, M.J.: Miettinen-Joensuu. *Covariables were: age, sex, adjuvant TKI therapy and mutational status for C1; and age, sex and mutational status for C2 and C3.

Figure S2 Kaplan-Meier curves for recurrence-free survival (RFS) and overall survival (OS) in the subgroup of C2 and C3 patients with high risk score according to pathological Miettienen-Joensuu scoring system and Imatinib sensitive mutations depending on the deep Miettinen-Joensuu models. Kaplan-Meier curves for RFS (A) and OS (B) depending on the deep Miettinen-Joensuu model employing the C2 DL Score in this subgroup of the C2 subcohort. Kaplan-Meier curves for RFS (C) and OS (D) depending on the deep Miettinen-Joensuu model employing the C3 DL Score in this subgroup of the C3 subcohort. *: p < 0.05; ***: p < 0.001. Tests are log-rank tests.

Supplementary Tables Supplementary Tables.

## Data Availability

The data analyzed in this study are available from the respective original collecting institutions. Restrictions apply to the availability of these data, which were used under license for this study. Data are available from the authors upon reasonable request with the permission of the institution that originated the specific requested dataset.

## Code Availability

The only original code from this study is the Grammar Curation Tool, available at https://github.com/KatherLab/GraDaCu under Massachusetts Institute of Technology license. The code was developed specifically for this study and does not include reused components from previously published repositories or software. All other analyses used previously published code.
